# A Unique and Extensive Constellation of Late Radiation Effects in a Single Patient Following High-Dose Radiation Therapy for Childhood Hodgkin’s Lymphoma

**DOI:** 10.7759/cureus.100928

**Published:** 2026-01-06

**Authors:** Jacob M Fryer, Erica B Fuller, Timothy B Dinh, David T Padro

**Affiliations:** 1 Diagnostic Radiology, Tripler Army Medical Center, Honolulu, USA; 2 Transitional Year Program, Tripler Army Medical Center, Honolulu, USA; 3 Nuclear Medicine, Tripler Army Medical Center, Honolulu, USA; 4 Radiation Oncology, Tripler Army Medical Center, Honolulu, USA

**Keywords:** childhood hodgkin's lymphoma survivorship, extended-field radiation therapy, humeral head osteonecrosis, multi-joint osteonecrosis, multiple secondary malignancies, radiation-induced cardiovascular disease, radiation-induced late effects, radiation-induced osteonecrosis, radiation-induced pulmonary artery calcification

## Abstract

Childhood cancer survivors treated with large-field radiation may develop late secondary effects across multiple organ systems. We present a 60-year-old male whose PET/CT revealed an absent thyroid gland with multiple metastatic foci, a surgically absent spleen and bladder with an ileal conduit, severe pulmonary outflow tract calcifications, and transcatheter aortic valve replacement (TAVR), bilateral proximal humeral osteonecrosis, and bilateral total hip arthroplasties. Though each individual finding may be readily recognizable on imaging, the key to this case is interpreting the constellation of findings as potential late effects of radiation therapy (RT), especially since severe pulmonary artery calcifications and multifocal osteonecrosis involving both shoulders and hips due to RT are exceedingly rare. Review of the patient’s medical history, obtained only after persistent follow-up with the treating facility, revealed prior high-dose, large-field external beam radiation for stage IIIA childhood Hodgkin’s lymphoma, administered in the 1970s. Awareness of these characteristic sequelae allows medical providers to correctly contextualize seemingly disparate findings, particularly when the details of prior radiation history are not readily available.

## Introduction

In the mid-to-late 20th century, high-dose (35-45 Gy) extended-field radiation therapy (EFRT) for childhood lymphoma was a common practice, encompassing both involved and uninvolved nodal regions with normal adjacent tissues [1]. Survivors face elevated risks for a broad range of long-term effects, including secondary malignancies, cardiovascular disease, and musculoskeletal complications, all of which are well supported by the existing literature [2-4]. However, to our knowledge, very few case reports mention severe radiation-induced pulmonary artery calcifications and multifocal major proximal joint osteoradionecrosis, particularly involving the humeral heads. Moreover, no case report has documented the concurrent presentation of the aforementioned rare findings with other uncommon radiation sequelae, including multiple secondary malignancies and severe cardiovascular disease resulting in aortic valve replacement.

## Case presentation

An F-18 fluorodeoxyglucose (FDG) PET/CT study was performed at our institution for a 60-year-old man referred for surveillance of stage IV papillary thyroid carcinoma following total thyroidectomy and multiple rounds of radioiodine therapy. Only limited historical information was available in the patient’s chart at the time of imaging.

Imaging findings

**Figure 1:**
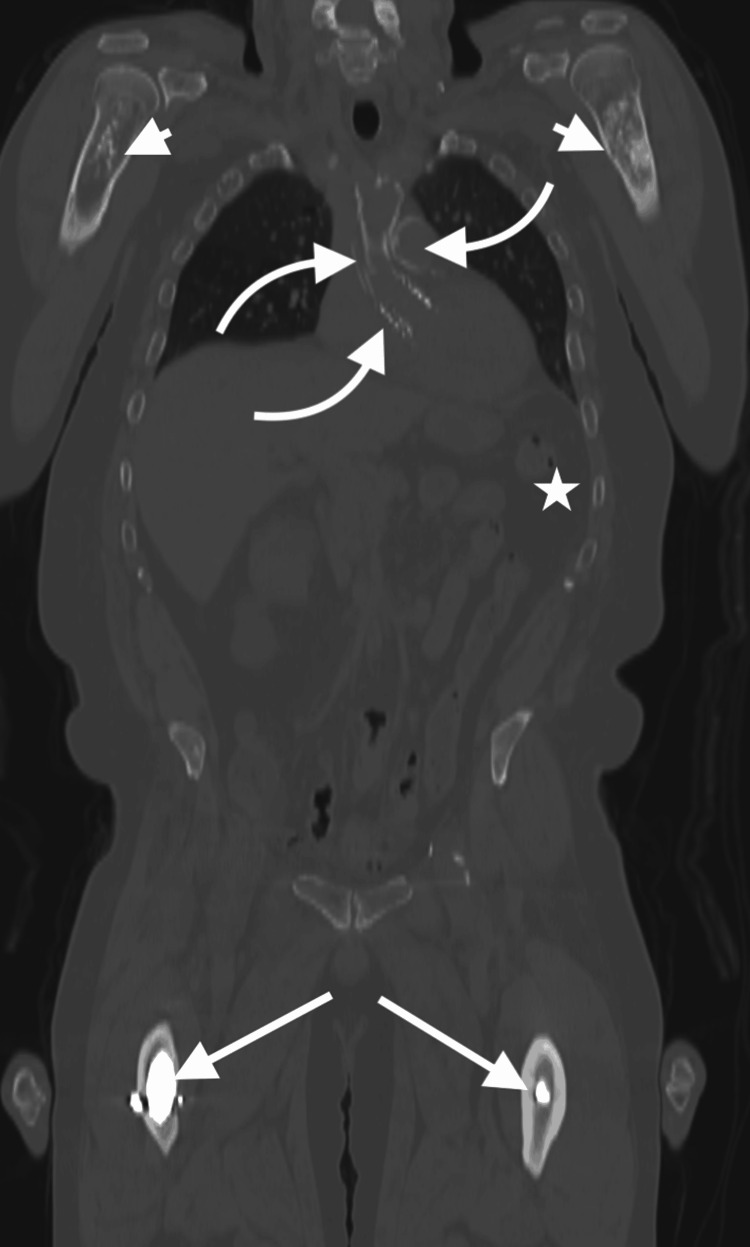
Coronal non-contrast CT in bone window from PET/CT. Demonstrates patchy sclerotic foci with scattered lucencies in the bilateral proximal humeri (short arrows), multiple mediastinal calcifications and metallic hyperdensities (curved arrows) better described on subsequent figures, absence of the spleen in the left upper quadrant (star), and incompletely visualized surgical hardware in the bilateral proximal femora (long arrows) consistent with total hip arthroplasty.

A large coronal view of the chest, abdomen, and pelvis provided the most comprehensive overview of the patient’s findings in a single image, effectively highlighting the widespread post-treatment changes that suggest a history of large-field radiation exposure (Figure 1). From this view, one can appreciate changes consistent with osteonecrosis in the bilateral proximal humeri, extensive mediastinal calcifications and metallic hardware, a surgically absent spleen, and bilateral total hip arthroplasties. 

**Figure 2:**
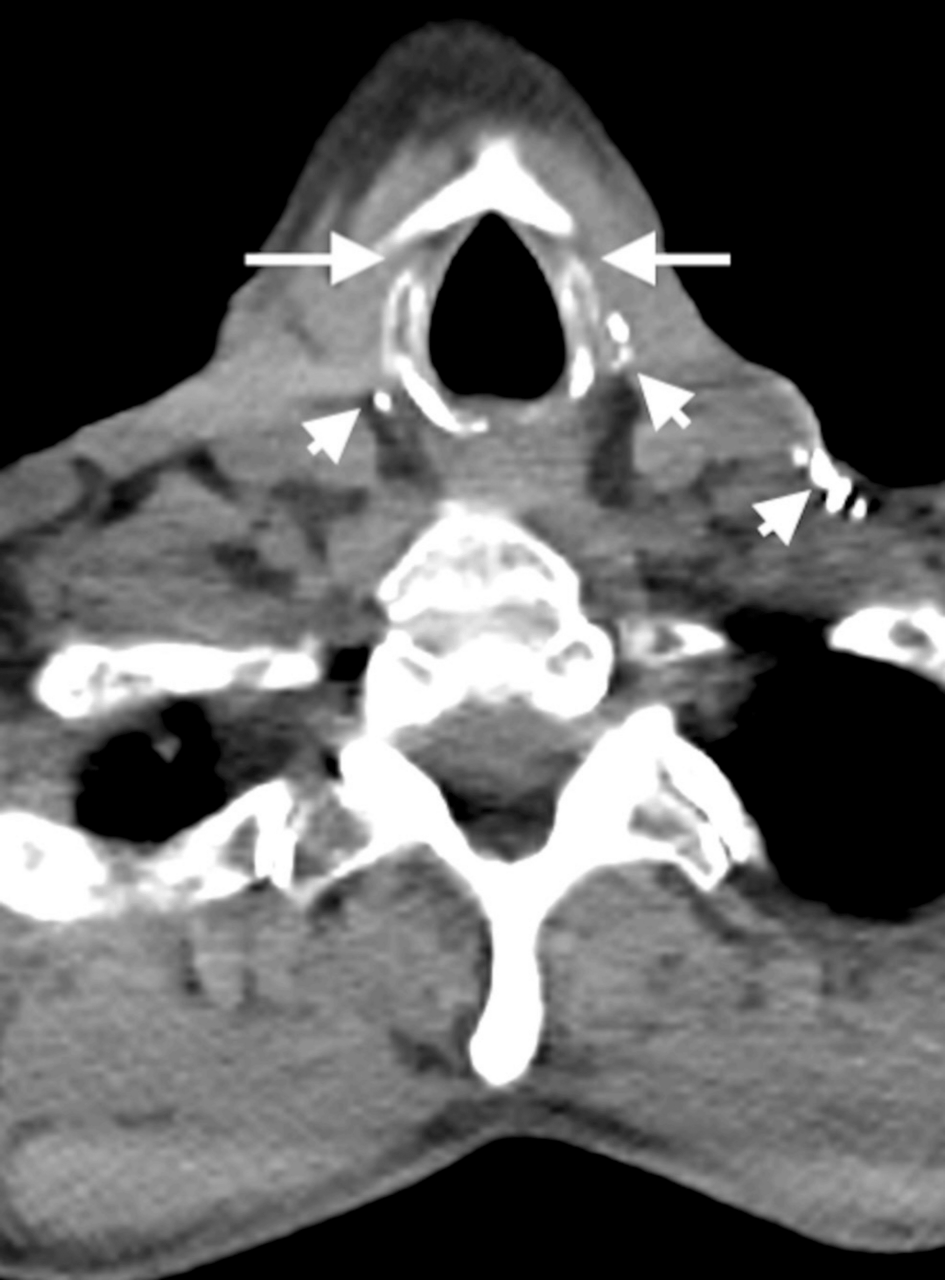
Axial non-contrast CT in soft tissue window from PET/CT through the level of the neck at the inferior thyroid cartilage. Demonstrates a surgically absent thyroid gland in the region of the thyroid bed (long arrows). The thyroid gland would normally appear in this area as a hyperdense butterfly-shaped soft tissue mass. Also noted in the anterior soft tissues of the neck are multiple surgical clips (short arrows) consistent with the patient’s history of total thyroidectomy and cervical lymph node dissection.

**Figure 3:**
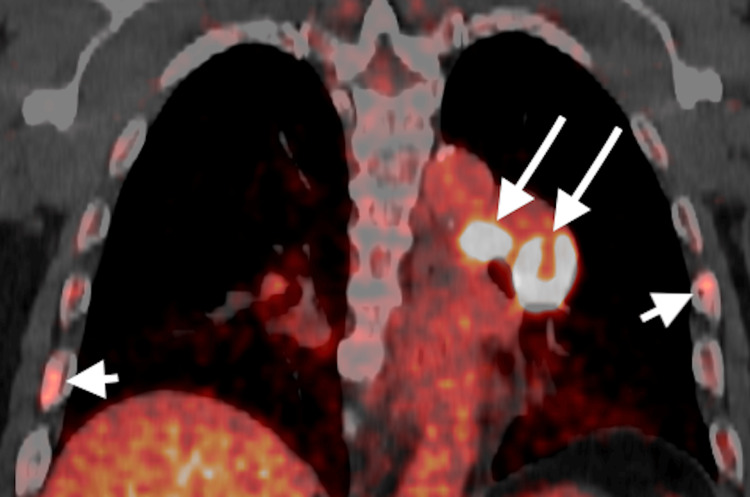
Reformatted coronal PET image through the chest. Demonstrates multiple FDG-avid foci involving the left perihilar region (long arrows) and bilateral ribs (short arrows).

**Figure 4:**
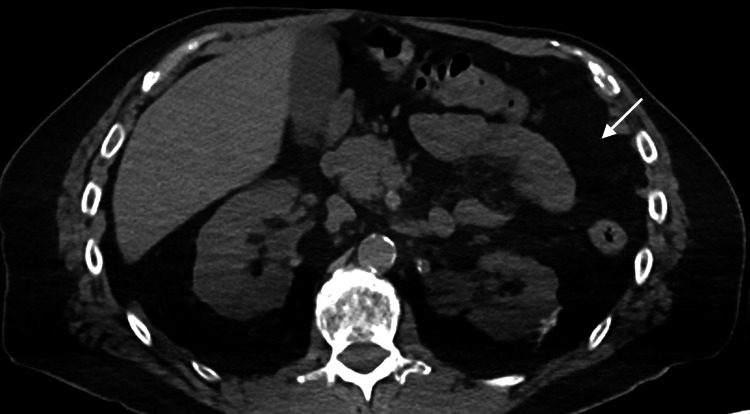
Axial non-contrast CT in soft tissue window from PET/CT through the level of the upper abdomen. Demonstrates absence of the spleen in the left upper quadrant (arrow).

**Figure 5:**
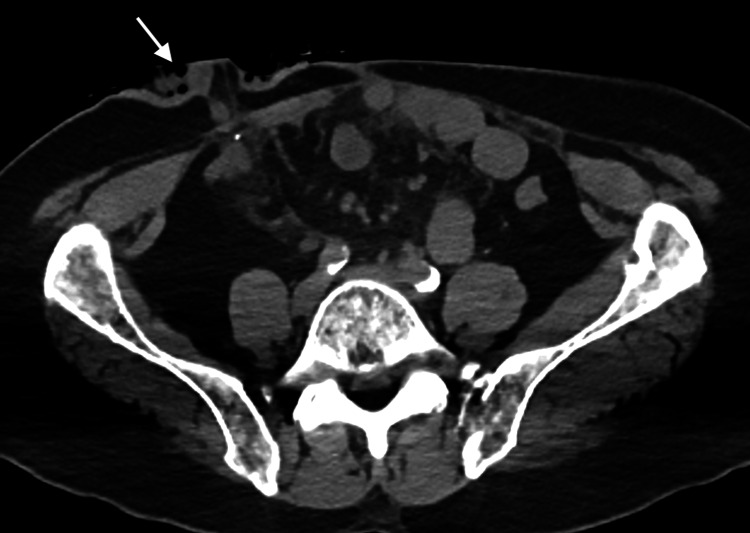
Axial non-contrast CT in soft tissue window from PET/CT through the level of the lower abdomen and pelvis. Reveals an ileostomy in the right lower quadrant (arrow), consistent with the patient’s history of ileal conduit after total cystectomy.

Further evaluation revealed a surgically absent thyroid gland (Figure 2) with multiple hypermetabolic perihilar and osseous metastases (Figure 3). The abdomen and pelvis again demonstrated a surgically absent spleen in the left upper quadrant (Figure 4) and a surgically absent urinary bladder with an ileal conduit (Figure 5). 

**Figure 6:**
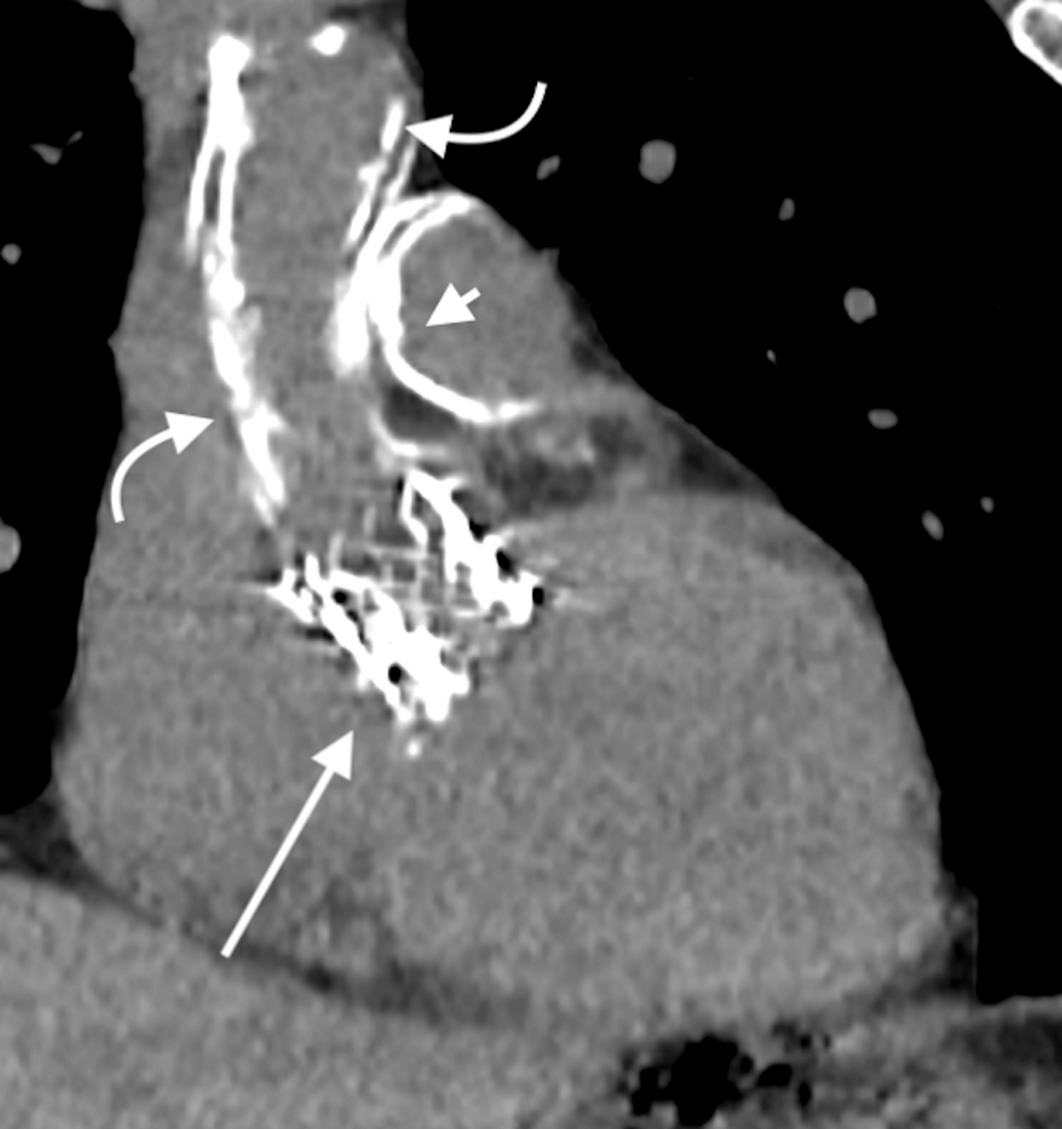
Coronal non-contrast CT in soft tissue window through the mediastinum. Shows severe atherosclerotic calcifications of the ascending aorta (curved arrows), a metallic mesh-like stent (long arrow) consistent with the patient’s history of TAVR, and severe calcifications of the pulmonary infundibulum and pulmonary valve (short arrow). TAVR: transcatheter aortic valve replacement

**Figure 7:**
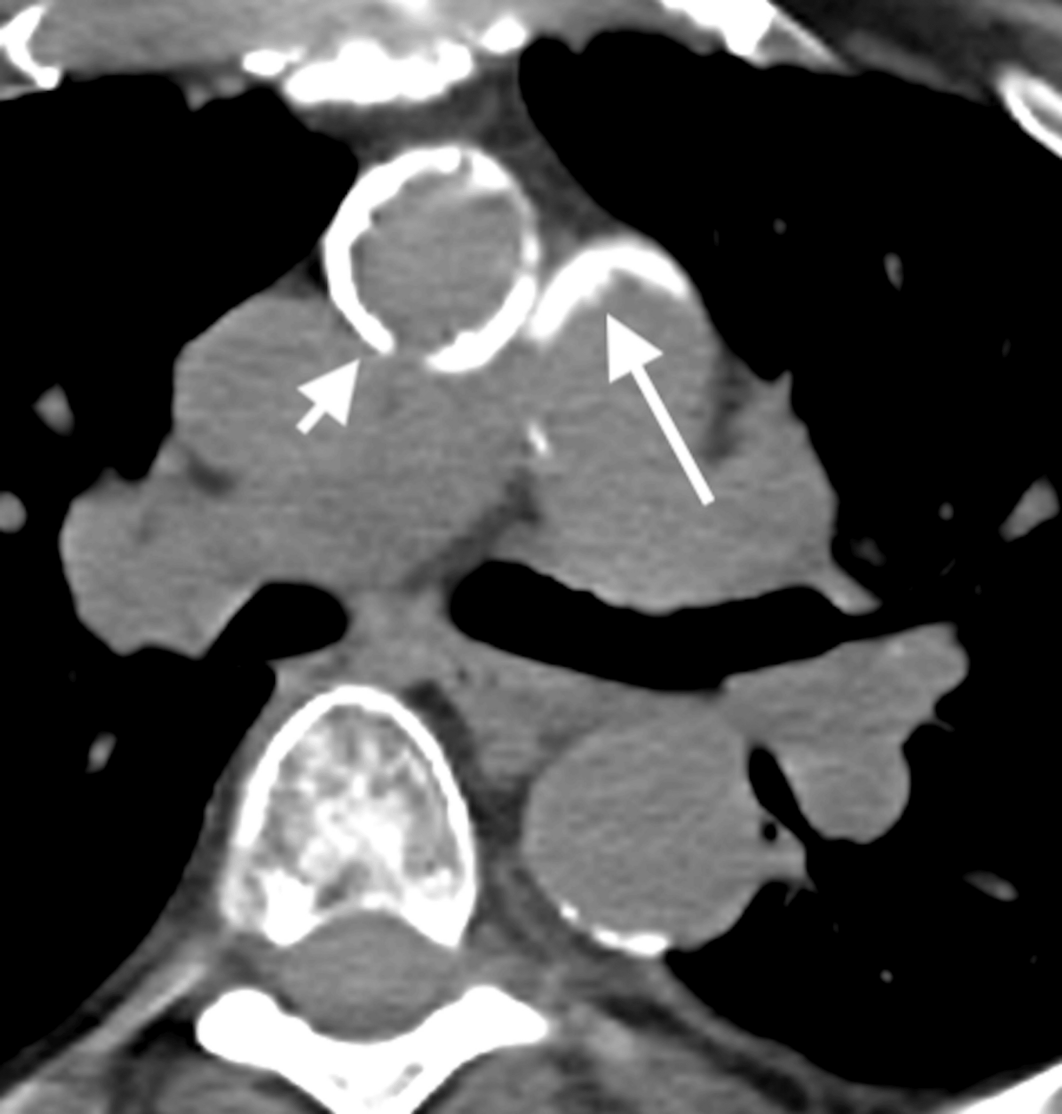
Axial non-contrast CT in soft tissue window through the mediastinum. Shows severe atherosclerotic calcifications of the ascending aorta (short arrow) and severe calcification of the pulmonary trunk (long arrow) with mild extension into the left main pulmonary artery.

There was severe atherosclerosis of the ascending aorta, multivessel coronary calcifications, and marked calcifications involving the pulmonary infundibulum, pulmonary valve, and pulmonary trunk, with extension into the main pulmonary arteries. Postsurgical changes were also consistent with transcatheter aortic valve replacement (TAVR) (Figures 6, 7). 

**Figure 8:**
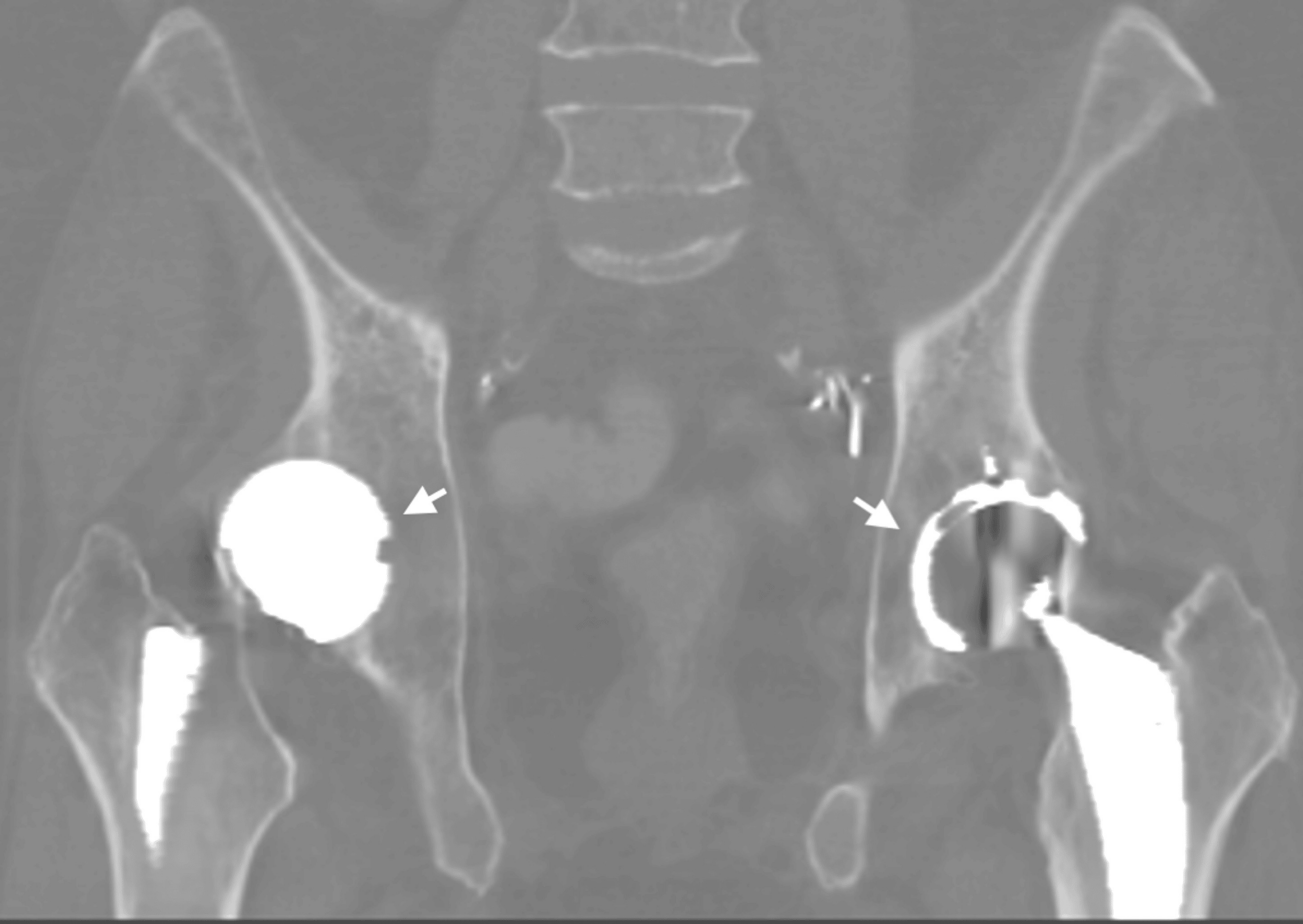
Coronal non-contrast CT in bone window through the pelvis. Demonstrates bilateral total hip arthroplasties (arrows) without complication.

**Figure 9:**
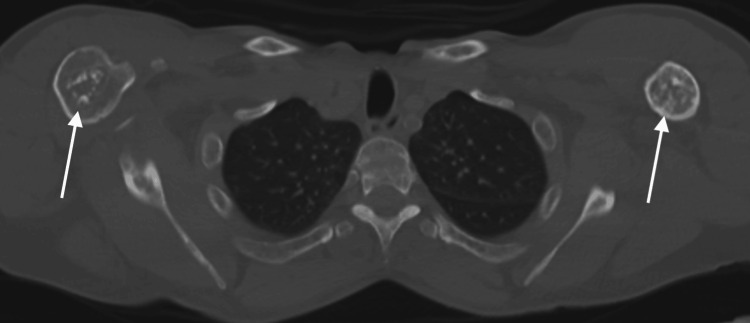
Axial non-contrast CT in bone window from PET/CT through the level of the upper chest. Demonstrates patchy sclerotic foci with scattered lucencies in the bilateral proximal humeri (arrows), concerning for avascular necrosis.

Lastly, assessment of the musculoskeletal system revealed bilateral total hip arthroplasties (Figure 8) in addition to sclerosis with patchy lucencies of the bilateral proximal humeri, most consistent with osteonecrosis (Figure 9). Taken together, these extensive post-treatment changes were striking in both their breadth and severity, prompting the radiologists to seek further clinical history. 

Clinical correlation

A review of the patient’s chart suggested a history of childhood lymphoma, though it was unclear whether radiation, chemotherapy, or a combination had been administered, and no documentation described the treatment fields, agents, or doses. The patient was advised to contact the hospital where he had received treatment as a child, but initial attempts to obtain records were unsuccessful. After several months of persistent follow-up, the historical records were eventually located. These confirmed that he had been treated in 1970 with Cytoxan and high-dose EFRT for stage IIIA Hodgkin’s lymphoma at age five. This included 35 Gy above and 35 Gy below the diaphragm using cobalt teletherapy. Shielding details were limited in the documentation. 

His medical and surgical history was also notable for multiple significant interventions over several decades. He underwent a splenectomy in 1969 for lymphoma involvement. Bilateral total hip arthroplasties were performed for early-onset osteoarthritis in 2003 and 2009. In 2013, he was diagnosed with papillary thyroid carcinoma, which was treated with total thyroidectomy followed by radioactive iodine therapy. 

In 2019, he developed urothelial carcinoma that required a total cystectomy with creation of an ileal conduit. Severe aortic stenosis was subsequently treated with TAVR in 2021. Most recently, in 2024, he was diagnosed with pulmonary hypertension attributed to severe pulmonary outflow tract calcification. 

## Discussion

Historical context

In the 1970s, EFRT was the standard of care for advanced-stage pediatric Hodgkin’s lymphoma [1,2]. Although our patient’s 35 Gy treatments were consistent with the era’s protocols [1,2], they unfortunately included large volumes of normal tissue. Modern National Comprehensive Cancer Network (NCCN) guidelines now recommend 21 Gy involved-site or involved-field RT (ISRT/IFRT), with advanced conformal planning to minimize toxicity [5]. For this reason, extensive late effects as seen in our patient are rarely seen with contemporary protocols. However, awareness remains crucial as survivors of historical regimens continue to present with their long-term sequelae.

Secondary malignancies, cardiovascular sequelae, and musculoskeletal complications

The occurrence of secondary malignancies following RT, including thyroid and bladder carcinomas, is well-documented [5,6]. The risk of developing secondary cancers increases with younger age at exposure, higher dose, and larger field size [5]. Thus, it is likely the patient’s secondary cancers represent well-documented late oncologic sequelae of high-dose EFRT in childhood [3,6]. Additionally, exposure to alkylating agents such as cyclophosphamide (Cytoxan) may have potentiated urothelial carcinogenesis, synergizing with radiation-induced DNA damage to the bladder [3,7]. 

The patient’s severe multivessel coronary calcification, aortic and pulmonary valve calcifications, and prior TAVR reflect radiation-induced cardiovascular disease (RICD), a known late complication of mediastinal irradiation [8]. RICD develops primarily from radiation-induced endothelial injury leading to chronic inflammation, fibrosis, and calcification of cardiac structures [8]. Cyclophosphamide, known to have cardiotoxic properties, may have compounded the associated findings via direct oxidative stress, interstitial fibrosis, and microvascular injury [7]. 

The bilateral humeral sclerosis with patchy lucencies and prior bilateral hip replacements is most consistent with radiation-induced osteonecrosis. Although it is rare among childhood cancer survivors, there is a significantly increased relative risk of developing osteonecrosis for those who received RT [9]. The American College of Radiology (ACR), moreover, lists radiation as a potential cause of avascular necrosis [10]. As discussed below, there are many alternative etiologies for osteonecrosis, but none were supported in our patient's case. 

Case novelty

The first exceptionally rare finding of this case includes extensive pulmonary outflow tract calcification with pulmonary valve dysfunction leading to pulmonary hypertension. According to the American Heart Association (AHA), there have been only five reports of pulmonary valvular stenosis with disease involving the entire pulmonary outflow tract after chest radiotherapy, and these cases only occurred at doses greater than 40 Gy, which is beyond what our patient was documented to have received [11]. One cross-sectional study assessing valvular disorders in 82 Hodgkin’s lymphoma survivors treated with mediastinal radiotherapy demonstrated a high prevalence of left-sided valvular disease, but pulmonary valve disease was not observed, implying that its prevalence is likely negligible [12]. The AHA explains that left-sided valvular disease is more frequent and pronounced compared to right-sided disease due to higher pressures of the systemic circulation [11]. 

The second novel aspect of this case is the patient’s multifocal osteoradionecrosis, particularly involving the shoulders. Multifocal major proximal joint osteonecrosis is present in the literature, but none, to our knowledge, were attributed to radiation. For example, three case reports present patients with concurrent shoulder and hip osteonecrosis, one of which was attributed to methylprednisolone, and the other two were deemed idiopathic [13,14]. Furthermore, the published literature documents only three individual case reports of unilateral humeral head osteoradionecrosis. More specifically, one case series of 19 shoulder arthroplasties for treatment of nontraumatic avascular necrosis of the humeral head found two cases occurring after radiation [15]. Another case series of 200 shoulders with osteonecrosis found only one attributable to radiation [16]. Additionally, in a large systematic analysis of 11,130 Hodgkin’s lymphoma survivors treated with radiation, there was a 1.2% incidence of avascular necrosis. Importantly, the study identified corticosteroids, rather than radiation, as the primary driver for these results, implying further rarity of osteoradionecrosis [17].

As alluded to above, one might reasonably argue that the patient’s multifocal osteonecrosis could have been attributed to other more common etiologies, such as corticosteroids. Steroid-containing chemotherapy regimens, after all, were included in treatment protocols of the era, such as cyclophosphamide, oncovin/vincristine, procarbazine, and prednisone (COPP) [18]. The patient’s chart, however, did not explicitly document receipt of COPP or other steroid-containing regimens, and no later records indicate corticosteroid use for subsequent cancers. The patient also denied any historical use of corticosteroids in addition to other potential causes of osteonecrosis, including alcohol abuse, major trauma, or hypercoagulable disease. Given this, the patient’s case represents a rare manifestation of multifocal osteonecrosis caused by RT, notably involving both shoulders. 

These rare findings on a background of other uncommon manifestations, including multiple secondary malignancies and severe aortic stenosis resulting in TAVR, contribute to the overall novelty of this case. For example, in a large cohort of 3,122 Hodgkin’s lymphoma survivors with a median 22.6-year follow-up, the cumulative incidence of a third malignancy (i.e., multiple secondary cancers) was 13.3% [19]. Another large cohort study demonstrates that the 30-year cumulative risk of moderate-to-severe valvular heart disease after mediastinal radiation is only 6.4% for a 31-35 Gy dose [20]. Although these findings are not necessarily rare, they are certainly uncommon, which further characterizes the overall uniqueness of our patient’s case in combination with the constellation of findings. 

Lastly, this case highlights an uncommon retrospective diagnostic pathway, where imaging findings prompted the discovery of a remote radiation history rather than confirming a known one. The patient’s PET/CT, performed for thyroid cancer surveillance, revealed an unusual constellation of findings without any mention of prior RT. Chart review revealed a history of childhood Hodgkin’s lymphoma but lacked treatment details. During tumor board discussion, radiologists and radiation oncologists considered historical large-field radiation as a possible unifying explanation, but the extent and severity of findings contributed to a significant level of uncertainty. Their investigation ultimately led to the retrieval of archival records from the 1970s, confirming treatment with high-dose cobalt teletherapy. 

Teaching points

Severe pulmonary outflow tract calcifications with pulmonary valve dysfunction leading to pulmonary hypertension and multifocal major proximal joint osteoradionecrosis, particularly involving the proximal humeri, are exceedingly rare radiation-induced late sequelae. 

The extensive multisystemic involvement of this case, spanning hematologic, endocrine, cardiovascular, genitourinary, and musculoskeletal systems, is exceptionally uncommon to encounter together in a single survivor, underscoring the importance of synthesizing cross-organ patterns rather than interpreting abnormalities in isolation. Awareness of this rare constellation of findings can prevent diagnostic fragmentation, particularly when details of historical treatment are unavailable, prompting radiologists and clinicians alike to consider prior large-field radiation as a unifying etiology.

Such broad late effects are now exceedingly rare due to modern field-limited radiation techniques, making historical cases valuable reference points for recognizing long-term survivorship complications. Our case serves as an important radiologic “time capsule,” reminding providers of the long-term cost of earlier treatment strategies and the importance of vigilant surveillance in aging childhood cancer survivors. 

## Conclusions

This case highlights how high-dose, large-field radiation in early childhood can lead to a lifelong cascade of multisystemic injury. The concurrent presence of secondary malignancies, cardiovascular calcifications, and multifocal osteonecrosis in a middle-aged adult should prompt radiologists to consider prior childhood radiation exposure, especially when such a history is not explicitly documented in the imaging request. 

Each abnormality in this patient could easily be misinterpreted in isolation, especially given the combination of rare and uncommon manifestations; however, recognizing their unifying etiology, radiation-induced late effects, allows for accurate and clinically meaningful interpretation. As modern treatment protocols continue to evolve, such extensive late sequelae will likely become increasingly less common. Nonetheless, the growing population of childhood cancer survivors means providers must remain vigilant in recognizing the imaging signatures of historical therapies. Integrating cross-system imaging findings with an understanding of past treatment strategies is essential to avoid diagnostic fragmentation and ensure comprehensive survivorship care.
